# Feasibility of Ecological Momentary Assessment of Daily Sexting and Substance Use Among Young Adult African American Gay and Bisexual Men: A Pilot Study

**DOI:** 10.2196/resprot.6520

**Published:** 2017-02-02

**Authors:** Sabrina L Smiley, Hoda Elmasry, Monica Webb Hooper, Raymond S Niaura, Alison B Hamilton, Norweeta G Milburn

**Affiliations:** ^1^ Schroeder Institute for Tobacco Research and Policy Studies at Truth Initiative Washington, DC United States; ^2^ Case Comprehensive Cancer Center School of Medicine Case Western Reserve University Cleveland, OH United States; ^3^ Department of Health, Behavior, and Society Johns Hopkins Bloomberg School of Public Health Johns Hopkins University Baltimore, MD United States; ^4^ Georgetown University Medical Center Lombardi Comprehensive Cancer Center Georgetown University Washington, DC United States; ^5^ Department of Psychiatry and Biobehavioral Sciences Semel Institute for Neuroscience and Human Behavior University of California, Los Angeles Los Angeles, CA United States; ^6^ Veterans Administration Greater Los Angeles Healthcare System Veterans Administration Center for the Study of Healthcare Innovation, Implementation, and Policy Los Angeles, CA United States

**Keywords:** ecological momentary assessment, mobile phone, text messaging, sexting, marijuana, alcohol, young adult, gay and bisexual, African American men

## Abstract

**Background:**

Recent evidence suggests that sexualized text communication (“sexting”) is associated with substance use and sexual risk behaviors among young adults, yet little is known about this relationship among young adult African American gay and bisexual men, a population disproportionately impacted by HIV in the United States. Rapid advances in mobile phone technology indicate a clear need for research using mobile health (mHealth) methods such as ecological momentary assessment (EMA) to serve as a viable counterpart to retrospective evaluation methods by using real-time data collection to assess sexting and substance use among this population.

**Objective:**

The objective of this pilot study was to (1) describe the EMA study design and protocol, (2) characterize the study population, and (3) assess the feasibility of a random prompt text message-based thrice-daily EMA over 14 days, as a means of prospectively studying sexting, marijuana, and alcohol use among a sample of young adult African American gay and bisexual men ages 21 to 25.

**Methods:**

Participants were recruited through flyers and snowball sampling during spring and summer 2015 at a community-based HIV/AIDS prevention, care, and support organization in Washington, DC. Eligible participants were enrolled in a one-time in-person study visit that consisted of informed written consent to participate in the study, a self-administered survey, a semi-structured interview, and enrollment and training in EMA data collection. Commencing the day after the study visit, a random prompt survey was texted to participants on their personal mobile phones 3 times a day over a 14-day data collection period assessing mood, texts sent, texts received, sexts sent, sexts received, marijuana want, marijuana use, and alcohol use.

**Results:**

EMA feasibility was tested with 25 self-identified African American gay (n=16) and bisexual (n=9) men (mean age of 23.48 years, SD 1.5). Each random prompt survey had 8 questions with responses including yes/no and Likert scale options. There were 104 total days of EMA observation, and the retention rate was 72% (18 out of 25 participants). Participants responded to the random prompt surveys with a 57.3% compliance rate providing a total of 544 completed surveys out of 949 surveys. The overall mean response time to complete a survey was 6.1 minutes. There were significant positive associations between EMA texts sent and received questions (ρ 0.84, *P*<.001) as well as sexts sent and received queries (ρ 0.72, *P*<.001).

**Conclusions:**

The use of an EMA protocol has the potential to be a very useful research tool for understanding episodic behaviors such as sexting and substance use in this relatively understudied and underserved population, and has implications for practice. Additional research is needed on how to maximize survey compliance.

## Introduction

Despite an overall reduction in new HIV infections in the United States, young African American gay and bisexual men continue to experience a disproportionate burden of HIV [1]. In 2014, among all African American gay and bisexual men diagnosed with HIV in the United States, an estimated 39% (4321) were aged 13-24 [1]. Young adulthood is a critical period of vulnerability for HIV-related risk behaviors including uptake in consumption of mood-altering substances before and during sexual encounters [2,3]. Among young adult African American gay and bisexual men, the most commonly used substances are alcohol and marijuana [4].

In recent years, researchers have begun to investigate an emerging mobile phone technology trend—sexualized text communication known as “sexting” [5-7]. Sexting describes sending and receiving sexually suggestive photos or text messages via mobile phone [5-7]. Benotsch et al found that sexting among young adults was associated with substance use (eg, alcohol, marijuana, ecstasy, cocaine), multiple sexual partners, unprotected sexual encounters, and sexually transmitted infections [5]. Bauermeister et al found that sexting was more prevalent among young adult gay and bisexual men compared to their heterosexual peers [7]. Although valuable contributions to the literature, both studies [5,7] were limited either by a predominately white, heterosexual or non-heterosexual sample, making it difficult to generalize to young adult African American gay and bisexual men.

Retrospective self-reports are the dominant method of data collection for assessing sexting and substance use among young adults [5-7]. However, collecting data through self-reporting methods can threaten the reliability and validity of measurement, particularly if individuals under- or overreport their behavior [8-10]. Ecological momentary assessment (EMA), a mobile health (mHealth) method that involves repeated sampling of an individual's behavior in real-time, is an innovative counterpart to traditional research methods that have heavily relied on retrospective self-reported measures [9,10]. EMA can increase the accuracy of self-disclosed, episodic behaviors such as sexting and substance use, and offers an observational longitudinal approach for collecting multiple, real-time assessments of these behaviors over time, and in an individual's environment [9,10]. The use of EMA also offers unique advantages, as it is designed to minimize recall bias, capture time-stamped data, and increase validity in daily life settings [9,10]. Additionally, EMA employs mobile technology data collection tools, including PDAs, Palm Pilots, and mobile phones [9,10]. Rapid advances in mobile phone technology and text messaging have vastly impacted communication and offer a promising approach to developing EMA methods for understanding how mobile phone technology trends, such as sexting, are associated with HIV-related risk behaviors.

Building on existing literature that has used EMA methods with young adult African American gay and bisexual men, the objective of this pilot study was to (1) describe the EMA study design and protocol, (2) characterize the study population, and (3) assess the feasibility of a text message–based thrice-daily EMA over 14 days, as a means of prospectively studying sexting, marijuana, and alcohol use among a sample of young adult African American gay and bisexual men aged 21 to 25. Feasibility was assessed in terms of random prompt text message survey response compliance, question response compliance, and response times. Retention was also assessed, and recommendations for future EMA research and implications for practice are presented.

## Methods

### Study Participants

Participants were recruited through flyers and snowball sampling during spring and summer 2015 at a community-based HIV/AIDS prevention, care, and support organization in Washington, DC. Prospective participants were screened by phone by a trained study coordinator to determine whether they met the study’s eligibility criteria, which included self-identifying as black/African American, self-identifying as gay or bisexual, being between the ages of 21 and 25, residing in the Washington, DC, metro area, being English-speaking, having a personal mobile phone and using it daily, having a mobile phone plan with unlimited text messages, and being able to travel to the designated study site.

### Study Procedures

All eligible participants were screened and enrolled until the target sample size of 25 was met, which is characteristic of EMA pilot studies [11-13]. Participants completed a one-time study visit that consisted of informed written consent, a self-administered survey, a semi-structured interview, and training in EMA data collection. The self-administered survey assessed sociodemographic characteristics (eg, age, education, annual income, and current employment status), social networking application behavior (eg, self-reported use of the social networking applications Facebook and Jack’d), text messaging behavior (eg, self-reported daily texting frequency, sending and receiving sexts), sexual history (eg, self-reported number of male partners in the past 12 months), substance use (alcohol, combustible and noncombustible tobacco products, and marijuana), and psychological state (eg, self-reported anxiety and depression symptoms using the Patient Health Questionnaire for Depression and Anxiety [PHQ-4]) [14].

### Ecological Momentary Assessment Data Collection

After participants completed the self-administered survey, the primary author registered their phones to receive the random prompt surveys, and participants were trained on how to respond to them via dummy surveys triggered to their phones. The EMA data collection system at the primary author’s institution initiated random prompt surveys 3 times a day for 14 days. On weekdays, surveys were delivered between 6:00 AM and 23:59 PM. On weekends, surveys were delivered between 9:00 AM and 23:59 PM. The EMA data collection system recorded the date and time it took the participant to respond to and complete a random prompt survey, and the date and time a survey expired. The survey expired after 15 minutes of inactivity. The EMA data collection system returned an error message to prevent skipping items or entering out-of-range values. Text messages reminding participants to complete the random prompt surveys were delivered to participants’ phones each day at 8:00 PM, and the primary author was available by office phone, work cell, and email to respond to participants’ inquiries and concerns.

**Figure 1:**
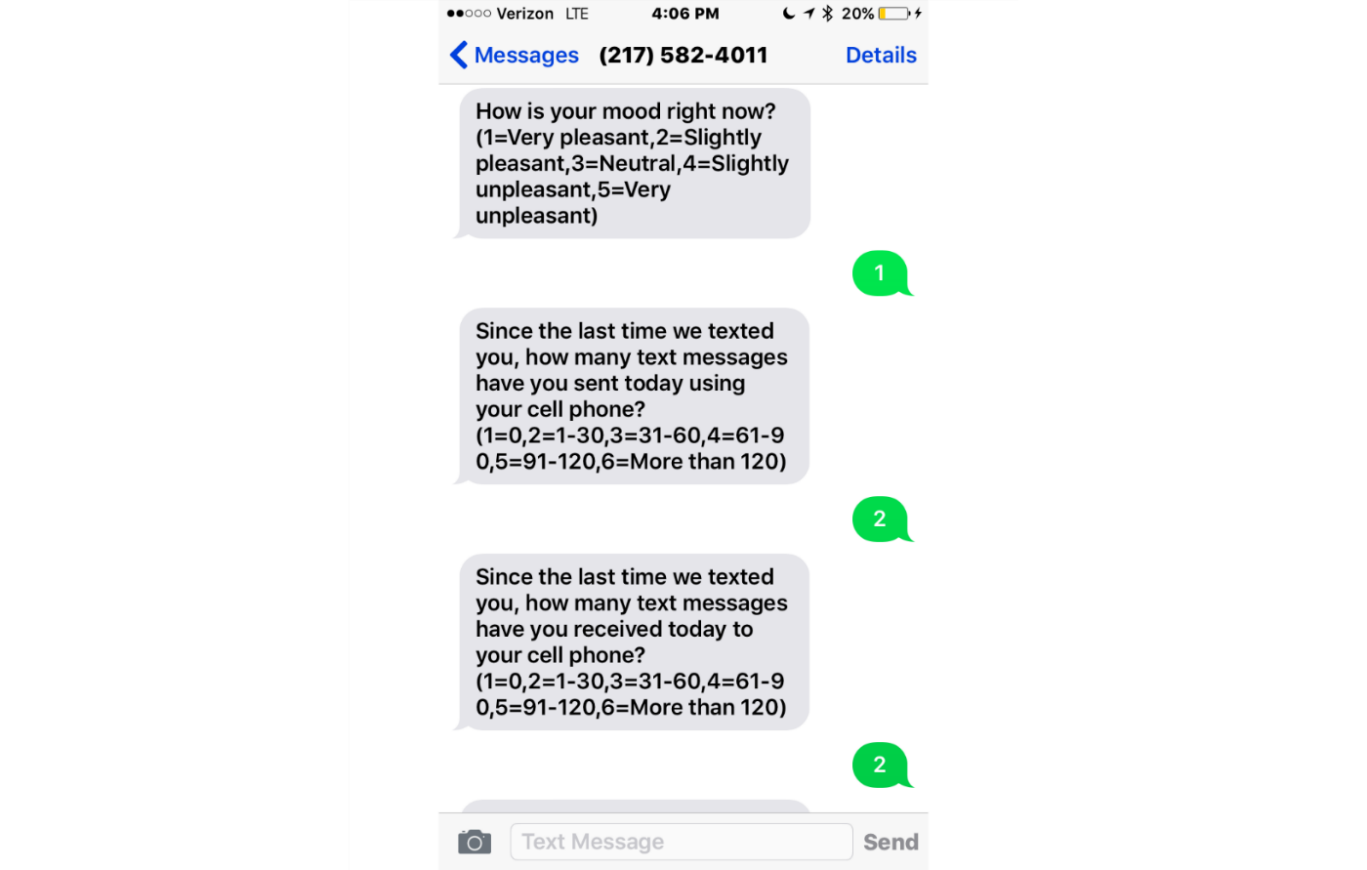
Screenshot of 3 of the 8 ecological momentary assessment (EMA) random prompt text message survey questions.

The random prompt survey consisted of 8 questions with responses including yes/no and Likert scale options. Questions were adapted from similar EMA questionnaires combined with current literature on sexting and substance use [7,15-17]. Figure 1 presents a screenshot of 3 of the 8 questions of the EMA random prompt text message survey. Questions included “How is your mood right now?” (1=very pleasant, 5=very unpleasant), “Since the last time we texted you, how many text messages have you sent today using your cell phone?” (1=0, 6=more than 120), “Since the last time we texted you, how many text messages have you received today to your cell phone?” (1=0, 6=more than 120), “Since the last time we texted you, did you send a sexually explicit message or photo of yourself using your cell phone today?” (1=yes, 2=no), “Since the last time we texted you, did you receive a sexually explicit message or photo of someone by way of cell phone today?” (1=yes, 2=no), “Right now, how much do you want to use marijuana?” (1=not at all, 4=to a great extent), “Since the last time we texted you, have you used marijuana?” (1=yes, 2=no), and “Since the last time we texted you, did you use alcohol, including sips of someone’s drink or your own drink?” (1=yes, 2=no).

Participants received a US $25 Visa gift card at the one-time study visit. Following 14 days of EMA data collection, participants who completed less than 50% (0-20) of the random prompt surveys received a US $25 Visa gift card for their time and cell phone usage. To maximize response compliance, participants who completed 50% or more (21-42) of the random prompt surveys received a US $50 Visa gift card. Additionally, during data collection, the primary author contacted participants via phone call, text message, or email and informed them of their progress toward earning the US $50 Visa gift card. The Chesapeake Institutional Review Board approved all study procedures (Pro00012060) and a Certificate of Confidentiality was obtained through the National Institute on Drug Abuse.

### Ecological Momentary Assessment Compliance Measures

Retention was defined as the percentage of participants who completed surveys on at least 10 of the possible 14 days of EMA data collection. Survey compliance reflects the proportion of complete surveys out of all surveys texted to the participant’s phone. To count as a complete survey, all 8 survey questions were required to be answered by the participant. Partial survey compliance includes abandoned surveys, where at least 1 survey question was answered but not all 8 survey questions. Question compliance is the proportion of questions answered out of all questions sent. Time to complete a survey reflects the time between the first survey prompt and when participants completed their responses. Participants were prompted 3 times at 5 minute intervals; as such, time to complete a survey reflects not only the actual time to answer all 8 survey questions but also the number of prompts sent before a response was received.

### Data Analysis

Descriptive statistics were used to characterize the study population in terms of sociodemographic characteristics, childhood experiences (parental smoking status, perceived introduction to adult responsibilities), self-reported health, personal financial status, use of social networks, sexualized text communication, sexual history, substance use, and compliance measures. Differences in survey compliance on baseline characteristics were examined using linear regression. Comparisons between week 1 and week 2 compliance measures were evaluated using paired *t* tests. Pairwise correlations were assessed for all 8 daily measures (mood, text sent, text received, sext sent, sext received, marijuana want, marijuana use, and alcohol use) using Pearson correlation. Cronbach alpha was also produced for the following related measures: (1) text sent, text received, sext sent, sext received and (2) marijuana want, marijuana use, alcohol use. All statistical analyses were completed in SAS version 9.4 (SAS Institute Inc) and Stata version 13 (StataCorp LP) with figures created in both SAS and JMP version 10.0.02 (SAS Institute Inc).

## Results

### Baseline Characteristics of Participants

Participants (n=25) were self-identified black/African American gay (n=16) and bisexual (n=9) men who ranged in age from 21 to 25 (mean 23.48, SD 1.5) years. More than half (15/25, 60%) reported annual incomes below US $35,000. The majority of the sample reported full or part-time employment (21/25, 84%) and at least some college education (21/25, 84%). When asked to describe their overall personal financial situation, 48% (12/25) of the sample reported just meeting basic expenses or not meeting expenses. In regard to childhood experiences, 64% (16/25) of the sample reported that one of their parents or guardians smoked cigarettes during their childhood. In terms of taking on adult responsibilities, 76% (19/25) of the sample reported growing up faster than other people their age. Most of the sample (19/25, 76%) reported using their phone multiple times per day to access social networking sites such as Facebook, Twitter, and Instagram. Nearly all (24/25, 96%) reported that they have received a sexually explicit photo of someone to their phone, and most of them (21/25, 84%) reported that they have sent a sexually explicit photo to someone using their phone. Over two-thirds (17/25, 68%) of the sample reported using marijuana every day or some days, and 56% (14/25) reported using little cigars/cigarillos/bidis at least 1 time in the past month. Additionally, 48% (12/25) reported drinking alcohol at least 2 to 3 times per week in the past month, and 36% (9/25) reported no condom use the last time they had anal sex with a man. According to the PHQ-4 [14], 52% (13/25) reported mild, moderate or severe psychological distress. Table 1 summarizes participants’ characteristics.

**Table 1:** Participant characteristics.

				Survey compliance	
				β	*P* value^a^
**Sociodemographics**					
	**Race, n (%)**				
		African American	21 (84)	5.72	.65
		More than 1 race (ref^b^)	4 (16)	—	—
	Age, mean (SD)		23.5 (1.5)	1.28	.68
	**Education, n (%)**				
		High school or less (ref)	4 (16)	—	—
		Some college or more	21 (84)	8.33	.51
	**Employment, n (%)**				
		Working, paid or unpaid	21 (84)	4.11	.75
		Not working (ref)	4 (16)	—	—
**Childhood**					
	**Parent/guardian smoked cigarettes, n (%)**				
		Yes	16 (64)	−14.53	.12
		No (ref)	9 (36)	—	—
	**Grew up compared to peers, n (%)**				
		Faster	19 (76)	−18.2	.08
		Slower/same rate (ref)	6 (24)	—	—
**Self-reported health**							
	**Health, n (%)**				
		Excellent or very good	17 (68)	11.66	.24
		Good or fair (ref)	8 (32)	—	—
	**Psychological distress, n (%)**				
		None (ref)	12 (48)	—	—
		Mild, moderate, or severe	13 (52)	−0.84	.93
**Personal financial status**					
	**Income, n (%)**				
		<US $35,000	15 (60)	—	—
		US $35,000+	8 (32)	—	—
		Unsure	2 (8)	—	—
	**Financial status, n (%)**				
		Live comfortably or meet needs with a little left	13 (52)	−8.11	.38
		Just meet basic needs or don't meet basic needs (ref)	12 (48)	—	—
	**Financial satisfaction, n (%)**				
		Pretty well satisfied or more or less satisfied	16 (64)	−12.46	.19
		Not satisfied at all (ref)	9 (36)	—	—
**Social networking technologies**					
	**Social networking on cell phone, n (%)**				
		Multiple times per day	19 (76)	−1.01	.93
		Daily, weekly, or never (ref)	6 (24)	—	—
	**Use Jack'd application, n (%)**				
		Multiple times per day or daily (ref)	6 (25)	—	—
		Weekly or monthly	12 (50)	4.78	.67
		Never	6 (25)	0.81	.95
**Sexting**					
	**Received sexually explicit text, n (%)**				
		Yes	24 (96)	—	—
		No (ref)	1 (4)	—	—
	**Received sexually explicit photo through text, n (%)**				
		Yes	24 (96)	—	—
		No (ref)	1 (4)	—	—
	**Sent sexually explicit text, n (%)**				
		Yes	23 (92)	—	—
		No (ref)	2 (8)	—	—
	**Sent sexually explicit photo through text, n (%)**				
		Yes	21 (84)	−8.90	.48
		No (ref)	4 (16)	—	—
**Sexual history**					
	**Used condom in most recent sexual encounter, n (%)**				
		Yes	15 (62.5)	22.71	.02
		No (ref)	9 (37.5)	—	—
**Substance use**					
	**Alcohol use past 30 days, n (%)**				
		2-4 times per month (ref)	13 (52)	—	—
		2-3 times per week	12 (48)	0.06	>.99
	**Marijuana use, n (%)**				
		Every day or some days	17 (68)	−6.30	.53
		Not at all (ref)	8 (32)	—	—
	**Little cigar/cigarillo use, n (%)**				
		Yes	14 (56)	−6.95	.46
		No (ref)	11 (44)	—	—

^a^*P* value for linear regression; regression analyses were not performed for variables with small cell sizes (n≤2).

^b^ref: referent.

### Feasibility Assessment

The EMA data collection period was programmed to last 14 days. There were 25 participants who provided 104 days of observation (April 17, 2015, through July 30, 2015). Average number of days of observation was 10.64 (SD 3.2, range 0-14). Due to a system scheduling error, 5 participants responded to the EMA random prompt text message surveys less than 14 days. Additionally, 1 participant’s phone was lost during the data collection period resulting in missing data. Another participant did not complete any surveys during the 14-day EMA data collection period but was included in the final sample since surveys were still sent to his phone. The total retention rate was 72% (n=18). Table 2 summarizes EMA compliance data.

**Table 2:** Ecological momentary assessment compliance data.

	Totals	Ranges
Number of participants, n (%)	25 (100)	
Total days of observation^a^, n	104	
Average days of observation per person, mean (SD)	10.64 (3.2)	Range: 0-14, interquartile range: 10-12
Retention^b^, n (%)	18 (72.0)	

^a^First entry 4/17/15, last entry 8/6/15 (when cut off at 2 weeks last day was 7/30/15, so 104 days).

^b^One participant lost phone and was unable to complete the study; retention defined as percentage of participants who completed surveys on at least 10 of 14 days.

Table 3 summarizes EMA compliance data overall and by week. A total of 949 random prompt surveys were texted to the sample’s phones and a total of 544 surveys were completed, resulting in an overall compliance rate of 57.3%. Due to the aforementioned system error, fewer surveys were sent out in week 2 compared to week 1. A total of 277/484 (57.2%) surveys were completed over week 1, and 267/465 (57.4%) surveys were completed over week 2. There were 41 surveys that were partially completed resulting in an overall partial survey compliance rate of 4.3%. Over week 1, 22/484 (4.5%) surveys were partial, and over week 2, 19/465 (3.9%) surveys were partial. Overall, 364 (36%) surveys expired due to nonresponse. A total of 4496 survey questions were completed resulting in an overall question compliance rate of 59.22%. Over week 1, 2290 (59.14%) questions were completed, and over week 2, 2206 (59.30%) were completed. Completion time was evaluated as the number of minutes elapsed from initiation of each survey to synchronization with the server. The overall mean response time was 6.06 (SD 4.99) minutes. The mean response time over the first week was 6.1 (SD 5.2) minutes, and over the second week it was 6.0 (SD 4.8) minutes.

**Figure 2:**
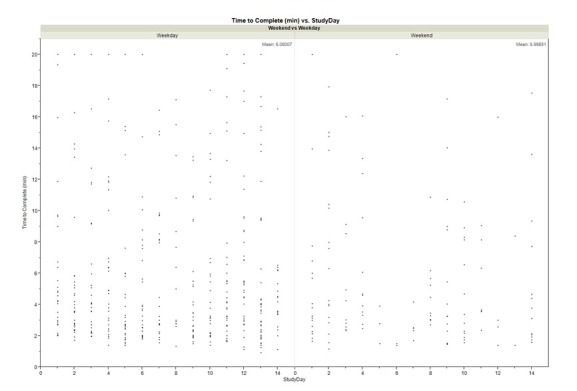
Time-to-complete survey from first prompt by system to last response by participant on weekdays versus weekends across study days.

Figure 2 shows the time, in minutes, participants took to complete the survey for each individual response on weekdays versus weekends across study days. Response time did not vary significantly between weekdays and weekends (M_weekday_: 6.1 [SD 5.0] minutes; M_weekend_: 6.0 [SD 5.0] minutes). Most points on weekdays and weekends lie beneath the 10-minute mark which indicates it took 2 or fewer system prompts to solicit a complete response from participants.

There were significant positive associations between EMA text sent and received questions (ρ: 0.84, *P*<.001) as well sexting sent and received queries (ρ: 0.72, *P*<.001). For text-focused EMA questions (text sent, text received, sext sent, sext received), the resulting Cronbach alpha was 0.68, suggesting acceptable internal consistency.

**Table 3:** Ecological momentary assessment compliance data overall and by week (data restricted to weeks 1 and 2 only).

		Overall	Week 1	Week 2
**Response/compliance rate, n (%)**				
	By survey^a^	544 (57.3)	277 (57.2)	267 (57.4)
	Partial surveys	41 (4.3)	22 (4.5)	19 (4.1)
	By question^b^	4496 (59.2)	2290 (59.1)	2206 (59.3)
**Status, n (%)**				
	Complete	544 (57.3)	277 (57.2)	267 (57.4)
	Expired	364 (38.4)	185 (38.2)	179 (38.5)
	Abandoned	41 (4.3)	22 (4.5)	19 (4.1)
Time to complete EMA^c^random survey^d^, mean (SD)		6.1 (5.0)	6.1 (5.2)	6.0 (4.8)

^a^Total number of random prompts completed/total number of random prompts possible (14 days × 25 participants × 3 surveys per day); excludes status code “initiating” from denominator.

^b^Total number of questions completed/total number of questions possible.

^c^EMA: ecological momentary assessment.

^d^Number of minutes elapsed from initiation of each random survey to synchronization with server; restricted to only complete responses and winsorized anyone who took over 20 minutes at 20 minutes.

## Discussion

### Principal Findings

To our knowledge, this is one of the first pilot studies to examine the feasibility of mobile phone-based text message EMA to prospectively capture sexting and marijuana and alcohol use among a sample of young adult African American gay and bisexual men. Study findings suggest that completing random prompt text message surveys 3 times per day across 14 days is a feasible approach. This study also underscores the importance of partnering with community-based HIV/AIDS prevention, care, and support organizations, as findings demonstrate the feasibility of recruiting, enrolling, and retaining young adult African American gay and bisexual men in an intensive, longitudinal pilot study.

This study found an overall survey compliance rate of 57.3% (544/949), with a rate of 57.2% (277/484) across the first week and a rate of 57.4% (267/465) across the second week. Previous EMA studies have reported compliance rates from 50% to 90% [12,18,19]. Compared to these studies, an overall survey compliance rate of 57.3% is promising, given that compliance was impacted by 8 days of observation due to the participant whose phone was lost during the data collection period. Additionally, data for the participant who did not complete any surveys—suggesting that he dropped out the day after his study visit—was included since random prompt surveys were still texted to his phone across the 14-day course of the study. Future studies will involve repeated testing of the system software and EMA protocol and procedures, to ensure quality assurance. Particular attention should be given to a lower-burden EMA protocol and procedures that better meet the needs of young adult African American gay and bisexual men. For example, each random prompt survey contained 8 questions. Since most participants (21/25) reported working either full-time or part-time, it is possible that they were less likely to complete a survey when they were engaged in the active duties of their employment. Future research will explore prompting participants to answer fewer than 8 questions in a survey.

There were also similar response rates in question compliance in the first week (2290/4496, 59.14%) compared to the second week (2206/4496, 59.30%). Future research will include conducting individual interviews or focus groups with members of the study population. These methods of formative research may provide an opportunity to explore why a participant may or may not complete a random prompt survey, thereby informing acceptability and strategies to maximize compliance.

Similar to other studies [15,20], participants were provided up to 15 minutes to complete a random prompt survey, and the mean time to complete across the 14-day assessment was 6.1 minutes. Findings demonstrate that participants completed surveys in a very timely manner. Additionally, mean response time on weekdays (6.1 minutes) and weekends (6.0 minutes) were all under 10 minutes, indicating that it took 2 or fewer system prompts to solicit a complete response (eg, answered all 8 questions in a survey) from participants. This suggests a high level of comfort with text messaging among participants. Mean time to complete a survey was slightly but not significantly faster on weekends, suggesting that participants may not have been as engaged in the active duties of their employment compared to weekdays.

### Implications for Practice and Future Research

In this study, nearly all participants reported having received a sexually explicit photo of someone to their phone (24/25, 96%) and having sent a sexually explicit photo to someone using their phone (21/25, 84%). Additionally, 68% (17/25) reported using marijuana every day or some days, 48% (12/25) reported drinking alcohol at least 2 to 3 times a week in the past month, and 36% (9/25) reported no condom use the last time they had anal sex with a man. The sexting, substance use, and sexual risk behavior among this sample of young adult African American gay and bisexual men suggests that EMA data collection tools, including mobile phones and text messaging, have the potential to address mobile phone technology trends such as sexting and reinforce substance use treatment and HIV prevention, care, and support services available through community-based HIV/AIDS organizations serving this population. Future research involves (1) repeated testing of the EMA protocol and data collection system, (2) limiting the number of questions asked in a random prompt text message survey, (3) soliciting buy-in to the study during orientation, (4) increasing study visits, (5) retraining participants during study visits, (6) providing feedback on response compliance, (7) providing a higher bar for incentives, (8) including both random prompts and self-reports to capture events and assess level of substance use, and (9) evaluating protocol acceptability by participants.

### Limitations

There are limitations to consider when interpreting study findings. While the sample size (n=25) was appropriate for pilot study research [21], the study is limited in generalizability, in that it uses a convenience sample of young adult African American gay and bisexual men who reported full or part-time employment, some college education, access to mobile phones with unlimited data plans, and residence in the Washington, DC, metro area. Although 15/25 participants (60%) reported annual incomes below US $35,000 and 9/25 participants (36%) reported that they just meet basic expenses, this study is unlikely to capture the diversity of viewpoints of all young adult African American gay and bisexual men. Additionally, in an effort to not overburden participants, EMA data collection was limited to 2 weeks. Future research should extend data collection to 3 or 4 weeks to provide more “in the moment” data.

### Conclusions

Compared to their white and Latino peers, young adult African American gay and bisexual men are arguably at an increased risk for HIV infection [1,22,23]. Mobile phone technology and text messaging present opportunities for novel, innovative research and HIV prevention strategies. This feasibility study highlighted the utility of an EMA approach for advancing knowledge about episodic behaviors, such as sexting and marijuana and alcohol use, to inform research and prevention strategies for this relatively understudied and underserved population. Additional research is needed to customize a fully effective EMA platform.

## Abbreviations

EMA: ecologic momentary assessment
PHQ-4: Patient Health Questionnaire for Depression and Anxiety
